# miR-342 Regulates BRCA1 Expression through Modulation of ID4 in Breast Cancer

**DOI:** 10.1371/journal.pone.0087039

**Published:** 2014-01-27

**Authors:** Elisabetta Crippa, Lara Lusa, Loris De Cecco, Edoardo Marchesi, George Adrian Calin, Paolo Radice, Siranoush Manoukian, Bernard Peissel, Maria Grazia Daidone, Manuela Gariboldi, Marco Alessandro Pierotti

**Affiliations:** 1 Department of Experimental Oncology and Molecular Medicine, Fondazione IRCCS Istituto Nazionale dei Tumori, Milan, Italy; 2 Molecular Genetics of Cancer, Fondazione Istituto FIRC di Oncologia Molecolare, Milano, Italy; 3 Institute for Biostatistics and Medical Informatics, Faculty of Medicine, University of Ljubljana, Ljubljana, Slovenia; 4 Department of Experimental Therapeutics and The Center for RNA Interference and Non-Coding RNAs, The University of Texas, M. D. Anderson Cancer Center, Houston, Texas, United States of America; 5 Unit of Medical Genetics, Department of Preventive and Predictive Medicine, Fondazione IRCCS Istituto Nazionale Tumori, Milano, Italy; 6 Scientific Directorate, Fondazione IRCCS Istituto Nazionale dei Tumori, Milan, Italy; Macquarie University, Australia

## Abstract

A miRNAs profiling on a group of familial and sporadic breast cancers showed that miRNA-342 was significantly associated with estrogen receptor (ER) levels. To investigate at functional level the role of miR-342 in the pathogenesis of breast cancer, we focused our attention on its “*in silico*” predicted putative target gene ID4, a transcription factor of the helix-loop-helix protein family whose expression is inversely correlated with that of ER. ID4 is expressed in breast cancer and can negatively regulate BRCA1 expression. Our results showed an inverse correlation between ID4 and miR-342 as well as between ID4 and BRCA1 expression. We functionally validated the interaction between ID4 and miR-342 in a reporter Luciferase system. Based on these findings, we hypothesized that regulation of ID4 mediated by miR-342 could be involved in the pathogenesis of breast cancer by downregulating BRCA1 expression. We functionally demonstrated the interactions between miR-342, ID4 and BRCA1 in a model provided by ER-negative MDA-MB-231 breast cancer cell line that presented high levels of ID4. Overexpression of miR-342 in these cells reduced ID4 and increased BRCA1 expression, supporting a possible role of this mechanism in breast cancer. In the ER-positive MCF7 and in the BRCA1-mutant HCC1937 cell lines miR-342 over-expression only reduced ID4. In the cohort of patients we studied, a correlation between miR-342 and BRCA1 expression was found in the ER-negative cases. As ER-negative cases were mainly BRCA1-mutant, we speculate that the mechanism we demonstrated could be involved in the decreased expression of BRCA1 frequently observed in non BRCA1-mutant breast cancers and could be implicated as a causal factor in part of the familial cases grouped in the heterogeneous class of non BRCA1 or BRCA2-mutant cases (BRCAx). To validate this hypothesis, the study should be extended to a larger cohort of ER-negative cases, including those belonging to the BRCAx class.

## Introduction

Breast cancer, the most common malignancy in women, is a heterogeneous disease exhibiting substantial diversity of histological and molecular characteristics that requires specific therapeutic interventions [1]. Most cases are classified as ‘sporadic’ breast carcinoma caused by genetic changes that occur over time [2], and only a small percentage (5–10%) is of hereditary origin. Mutations in BRCA1 and BRCA2 genes are the major cause of the hereditary form of the disease. The two genes may account for less than 30% of the genetic information responsible for heritable breast cancer, indicating that other genes could have a role in hereditary tumors with no BRCA1 or BRCA2 mutations, the so-called non-BRCA1/2 familial tumors (BRCAx) [3], [4], [5], [6].

In the last few years, microRNA (miRNA) expression profiling has emerged as a useful tool to stratify tumors and pinpoint miRNAs involved in specific steps of cancer progression [7], [8], [9]. These tiny, non-coding RNAs of 18–25 nucleotides in length, function as post-transcriptional regulators of gene expression by binding the 3′ untranslated region (UTR) of target mRNAs and promoting mRNA degradation or translational repression [10]. Several studies demonstrated that miRNAs can regulate the expression of oncogenes and tumor suppressors [11]. In breast cancer, miRNA expression profiling using microarray technology in association with some biopathological features such as estrogen receptor (ER), progesterone receptor (PR) and epidermal growth factor receptor 2 (HER2) status has been established as a useful tool to classify tumors [12], [13], [14], [15]. As ER, PR and HER2 are the molecular biomarkers currently used in routine clinical practice to help treatment decision in breast cancer, the identification of miRNAs whose expression could be related to their status could help to discover new molecular alterations involved in this tumor type [16].

In this study, we analyzed miRNA expression profiling of a group of heredo-familial and sporadic breast cancers. The classification of the samples according to their estrogen receptor status highlighted a strong association of ER with miR-342 expression levels. Further investigations led us to study ID4, a putative target of this miRNA, and its interaction with BRCA1. After demonstrating this mechanism in an *in vitro* model, we hypothesized that in our cohort of cases it could be involved in the pathogenesis of breast cancer, in particular in BRCAx familial cases.

## Materials and Methods

### Ethics Statement

This study was approved by the Independent Ethical Committee of the Fondazione IRCCS Istituto Nazionale Tumori of Milan (INT), and each patient provided written informed consent to donate the tissues left over after diagnostic procedures for research purposes.

### Breast Cancer Specimens

The study analyzed fifty-two specimens from 51 patients with breast cancer; the specimens were collected at INT during the period 1983–2003. The samples included 12 sporadic breast cancers (patients with a negative family history and age of onset >40 years) and 40 specimens with familial breast cancer. For one patient that developed bilateral disease both tumors were available and analyzed. All familial patients had early onset and/or positive family history, matched criteria for BRCA1 and BRCA2 molecular analysis used at INT [17], and did not overlap with any other known hereditary cancer syndromes. All familial patients underwent genetic counseling, with pedigree reconstruction going back for at least three generations, and were offered genetic testing (see Table S1 for features of cases). Tumor specimens containing more than 80% neoplastic cells were selected by an experienced pathologist from cryopreserved samples. Table S1 summarizes the clinical and histopathological features of the analyzed samples.

ER and PR status was routinely evaluated at time of diagnostic procedure according to the EORTC recommendations and within national [18] and international quality control programs by a ligand binding assay [19] and expressed as fmol mg^−1^ of protein. Tumors with an ER concentration higher than 10 fmol mg^−1^ of protein or with a PR concentration higher than 25 fmol mg^−1^ of protein were defined as ER-positive or PR-positive, respectively. HER2 status was immunohistochemically assessed with polyclonal antibody against p185 HER2 protein (1∶2000 dilution, DAKO) and defined as positive when strong membrane labeling was observed (2+ and 3+).

### Cell Lines

BT20 and MDA-MB-231 cells were obtained from the American Type Culture Collection; HCC1937 from DSMZ (Deutsche Sammlung von Mikroorganismen und Zellkulturen, Braunschweig, Germany); MCF7 and T47D cell lines were derived from the collection available at IFOM (Fondazione Istituto FIRC di Oncologia Molecolare, Milano); 293T from ICLC (Interlab Cell Line Collection, Istituto Nazionale per la Ricerca sul Cancro, Genova). Cells were tested and authenticated using the StemElite ID System (Promega). Each cell line was grown in a specific medium: BT20 in DMEM +10% fetal bovine serum; MDA-MB-231 in RPMI +5% fetal bovine serum; HCC1937 in RPMI +15% fetal bovine serum; MCF7 in MEM +10% fetal bovine insulin +0.01 mg/ml+1% NEAA (MEM Non Essential Amminoacids) +1% Sodium Pyruvate; T47D in DMEM +10% fetal bovine serum; 293T in DMEM +10% fetal bovine serum. All cell lines were maintained as a monolayer in a humidified incubator at 37°C with a supply of 5% CO_2_.

### miRNA Expression Analyses

Total RNA was extracted from tissue samples using Trizol (Life Technologies) and DNase I treated (Quiagen) according to the manufacturer’s protocol.

MicroRNA microarray profiling was performed for the 52 samples as described in [11], [20]. Briefly, 5 µg of total RNA was labeled and hybridized to each microRNA microarray containing 368 probes, including 245 human and mouse miRNA genes, in triplicate. Scanner images were quantified by the Quantarray software (Perkin-Elmer). Poor signal quality of background-corrected intensities were flagged and removed from the analysis, all intensities below 200 were thresholded to the value of 200; the expression values were log2 transformed and a Lowess normalization [21] was applied to each slide, using the internal replicates for the normalization. The normalized log2-tranformed expression ratios (sample/reference) of each miRNA were averaged and the arrays were median centered. Only human miRNAs (hsa-miR) were used for further analyses. The miRNA data sets were filtered by removing miRNAs with more than 50% missing (invalid) values. MiRNAs expression data have been submitted to the Gene Expression Omnibus (GEO) with accession number GSE46966.

### Quantitative Real-Time Polymerase Chain Reaction (qRT-PCR)

The RNA expressions of ESR1, PGR, ERBB2, BRCA1 and ID4 were measured by qRT-PCR on 1 µg of the same RNA used for miRNA analysis and on 1 µg of RNA extracted from the breast cancer cell lines with the same procedures used for the tissue specimens. Total RNA was reverse-transcribed using the High-Capacity cDNA Archive Kit and TaqMan reactions were performed in duplicate using TaqMan Assays-on-Demand Gene Expression Products (ESR1 Hs00174860_m1, PgRHs00172183_m1, HER2 Hs00170433_m1, ID4 Hs00155465_m1, BRCA1 Hs00173233_m1, Applied Biosystems). For miR-342 expression analysis, 30 ng of total RNA in a final volume of 15 µL was reverse-transcribed to cDNA using a High-Capacity cDNA Reverse Transcription Kit and miR-342 specific primers (Applied Biosystems) according to the manufacturer’s instructions. RT-qPCR was conducted using FAST chemistry (Applied Biosystems) in ABI PRISM 7700 and 7900 HT Real-Time PCR system (Applied Biosystems). To normalize data, 18S ribosomal subunit and GAPDH were used as housekeeping (4319413E, Hs99999905_m1, Applied Biosystems). The expression values of the miR-342 (002260) were normalized to RNU6B (001093). Data analysis was performed using the Sequence Detector v1.9 software.

### Identification of miRNAs Associated with Estrogen, Progesterone and Human Epidermal Growth Factor Receptor 2 Expression and Status

In order to identify the miRNAs associated with the estrogen receptor we fitted a linear regression model of each miRNA, using the normalized intensity of the miRNA as the outcome and the (log2-transformed) ESR1 expression (measured using qRT-PCR) as a covariate; similarly, we used the estrogen receptor status (ER-positive or ER-negative) as a covariate to identify the miRNAs associated with the ER status. We identified the miRNAs associated with progesterone and epidermal growth factor 2 receptors expression/status using similar models. The associations were considered statistically significant if the P values were less than 0.01.

To adjust the analysis for the presence of a possible batch effect in the miRNA experiment, all miRNA analyses were adjusted to the day on which the experiment was performed.

### Evaluation of the Association of BRCA1 with Genes Involved in Breast Cancer (ID4, PGR, ERBB2, ESR1) and miR-342

Using patients’ samples, we examined the association of BRCA1 gene expression with the expression of ID4, PGR, ERBB2, ESR1 and miR-342. The association was assessed fitting a multivariable linear regression model in which BRCA1 expression was the outcome variable and the expression of the genes and of miR-342 were the covariates; we also included an interaction term between the ER status and miR-342. The expression of the genes was evaluated using qRT-PCR, while the expression of miR-342 was measured on the miRNA arrays.

### Independent Case Series Used to Validate the Results

Publicly available data on mRNA and miRNA expression of breast cancers were retrieved from The Cancer Genome Atlas (TCGA) [22].

mRNA expression levels of BRCA1, ID4, ESR1, PGR and ERBB2, as well as ER and PR status, were available for 526 breast cancer patients; for 288 of them miR-342 expression was also available. Fifteen patients were BRCA1-mutant (12/15 with ER-positive status) and 22 were BRCA2-mutant (15/21 with ER-positive status). Family history of patients was not available, therefore all the other samples were classified as sporadics (384/485). Twenty two out of the 228 patients for which the information was available had an amplification of ERBB2.

### Computational Aspects

All the analyses were performed using BRB-ArrayTools v3.1 developed by Dr Richard Simon and Amy Peng Lam and R language for statistical computing (Development Core Team (2009). R: A language and environment for statistical computing. R Foundation for Statistical Computing, Vienna, Austria. ISBN 3-900051-07-0, URL. Available online at: http//www.R-project.org. Accessed November 24, 2012).

### Dual Luciferase Assays

The 3′ UTR region of ID4 gene (471 base pairs of length) containing the predicted miR-342 binding site was amplified by using the following primers:

For 5′-TCTAGATTTGTGACCAAGGAGCTCAA-3′,

Rev 5′-TCTAGATGCAATCATGCAAGACCACT-3′.

The PCR product was then digested with XbaI enzyme and cloned into the reporter plasmid pGL3 Promoter (Promega) downstream of the luciferase gene.

For reporter assays, 5×10^4^ 293T cells were seeded in 24 well plates and grown for 24 h. Subsequently, 0.1 µg of the pLuc ID4 3′ UTR construct together with 0.01 µg of Renilla luciferase pRLTK plasmid (Promega) were cotransfected with 100 nM of pre-miR-342 (mature sequence: UCUCACACAGAAAUCGCACCCGU, ID:PM12328) or pre-miR-1 (mature sequence: UGGAAUGUAAAGAAGUAUGUAU, ID: PM10617) or with pre-miR negative control#1 (Pre-miR-TM miRNA Precursor Molecule, Negative Control #1, ID: AM17110; Ambion) using Lipofectamine 2000 (Invitrogen) according to the manufacturer’s instructions. After 24 hours, the luciferase activity was measured using the Dual Luciferase Assay kit (Promega). Luciferase activity was normalized to Renilla activity to control the transfection efficiency.

### Transfection of miR-342 and miR Negative Control Precursor Molecules

The day before transfection, MDA-MB-231 and HCC1937 cells were seeded at a density of 25×10^4^ in 60-mm dishes. Pre-miR-342 precursor molecule and pre-miR negative control#1 (scramble) were transfected at a final concentration of 100 nM using Lipofectamine RNAiMAX (Invitrogen) according to the manufacturer’s instructions. MCF7 cells were seeded at a density of 35×10^4^ in 60-mm dishes and transfected with Oligofectamine (Invitrogen) according to the manufacturer’s instructions.

### ID4 Silencing

MDA-MB-231 cells were transfected with a siRNA pool of four oligonucleotides (ON-TARGETplus SMARTpool) targeting different portions of ID4 gene (ThermoScientific Dharmacon), at a final concentration of 25 nM using Lipofectamine RNAiMAX (Invitrogen). After 24 hours these cells were transfected with 100 nM of pre-miR-342 precursor molecule and collected 48 hours after the second transfection for RNA extraction and protein lysates.

### Western Blotting

Western blots were carried out for ID4 and BRCA1 protein. Seventy two hours after transfection with miR-342 precursor or negative control, MDA-MB-231, HCC1937 and MCF7 cells were collected by trypsinization and resuspended in 300 ul of 1X SDS sample buffer (62.5 Mm Tris-HCl pH 6.8, 2% w/v SDS, 10% glycerol, 50 mM DTT), supplemented with protease inhibitors cocktail (Calbiochem).

Protein quantification was performed using the BCA protein assay (Thermo Scientific). Forty micrograms of protein extract were loaded on 7% to 15% polyacrylamide gels and transferred to nitrocellulose membrane. The following primary antibodies were used: anti-ID4 (82-12, dilution 1∶1000, Calbioreagents), anti-BRCA1 (dilution 1∶800, Cell Signaling Technology), anti-β-Actin (dilution 1∶5000, MP Biomedicals), anti-Vinculin (dilution 1∶5000, Sigma-Aldrich) and anti-α**-**Tubulin (dilution 1∶5000, Sigma-Aldrich). Signals were detected using enhanced chemiluminescence system (Thermo Scientific).

Protein level quantification was performed using the software Imagelab©, by subtracting the global background of each membrane from the volume of intensity of the antibody detection bands (rectangle encompassing the whole band in the volume tools). The level of a protein in a certain sample was measured as the ratio between the quantifications of the band for the specific-antibody and the band for loading control.

## Results

### miRNAs Expression Profile Analysis

We obtained the miRNA profiles of 52 breast cancer tumors through hybridization on a custom microarray platform already validated by numerous studies [11]. The 52 samples included 15 positive cases for mutations of BRCA1 gene (BRCA1-mutant cases), 10 positive cases for mutations of BRCA2 gene (BRCA2-mutant cases), 15 cases with evidence of hereditary predisposition to breast cancer but without pathogenic mutations in BRCA1 or BRCA2 genes (BRCAx cases) and 12 cases not eligible for BRCA testing and with negative familial history (sporadic cases). Their main characteristics are reported in Table S1.

We evaluated the association of the miRNA profiles with estrogen, progesterone and human epidermal growth factor 2 receptors, measured both at mRNA expression levels (defined as ESR1, PGR and ERBB2), obtained by qRT-PCR, and at protein levels (defined as ER, PR and HER2) and classified positive or negative (status) as described in materials and methods. Regarding mRNA expression, we identified 22 miRNAs significantly associated to ESR1, 12 miRNAs associated to PGR and 11 related to ERBB2 levels (P<0.05). Dividing samples according to their protein levels for the three studied markers (status), 14 miRNAs were associated to ER, 5 to PR and 4 to HER2 status (P<0.05) (Table S2 and Figure S1).

**Figure 1:**
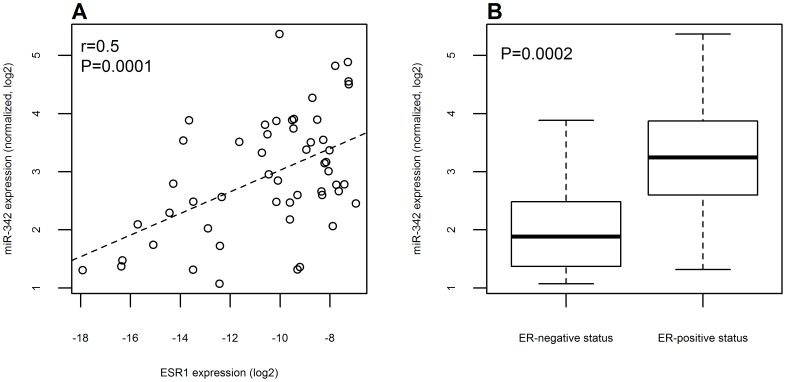
miR-342 expression in patients according to ESR1 expression levels or ER status. (A) Positive correlation of log2 normalized expression of miR-342 detected on miRNA arrays and log2 transformed ESR1 levels measured by qRT-PCR in the patients analyzed. The dashed line was obtained using a linear regression model. (B) Box plot representation of miR-342 expression according to estrogen receptor negative or positive status of the patients analyzed.

miR-342 had the strongest positive correlation with ESR1 expression (Pearson correlation r = 0.5, P = 0.0001, Figure 1A) and had the biggest average difference between patients with ER-positive status and those with ER-negative status (Figure 1B and Figure S1). We estimated that patients with ER-positive status had on average 2.1 times higher miR-342 expression compared to patients with ER-negative status (P = 0.0002, two-sample t-test, Figure 1B). A very similar association between ER and miR-342 was observed on the TCGA data set (Figure S2).

### miR-342 Directly Targets ID4

In three public databases for miRNA targets prediction (microRNA.org, release August 2010; TargetScan 6.2 and PicTar, release March 2007), we searched for putative 3′UTR gene targets of miR-342. We focused on the ID4 gene that was a predicted target of miR-342 in all the three databases. ID4 belongs to a family of dominant negative regulators of basic helix-loop-helix transcription factors and is highly expressed in diverse cancer types, including breast cancer [23]. The expression of this gene has also been found inversely correlated with that of ER status [24].

**Figure 2:**
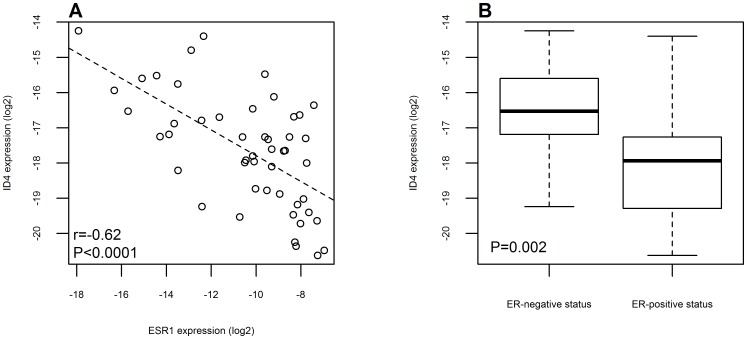
ID4 expression in patients according to ESR1 expression levels or ER status. (A) Negative correlation of ID4 expression and ESR1 levels both measured by qRT-PCR analysis. The dashed line was obtained using a linear regression model. (B) Box plot representation of ID4 expression considering estrogen receptor negative or positive status of the patients analyzed.

**Figure 3:**
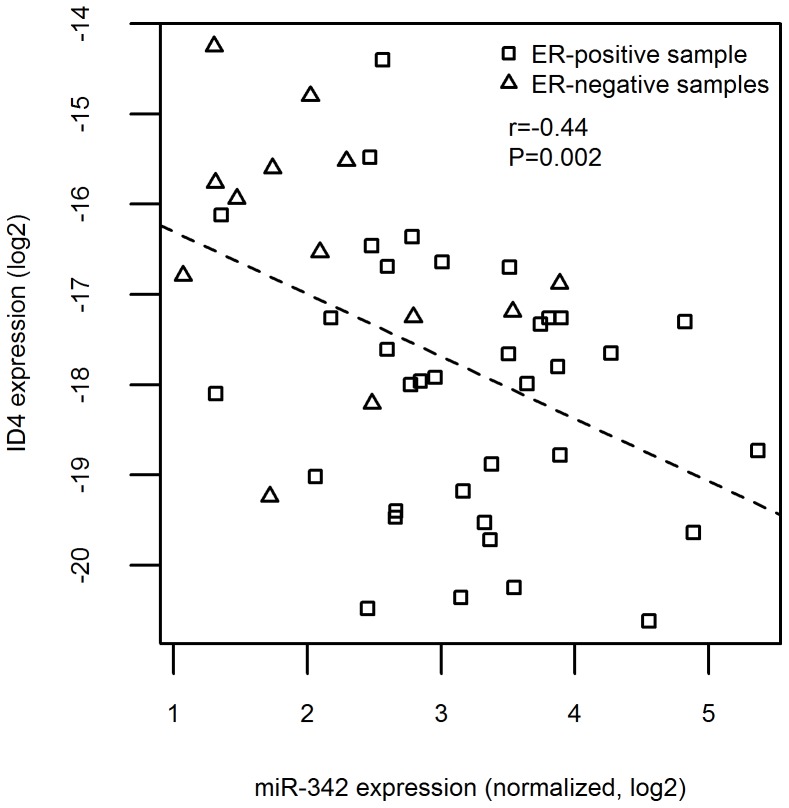
Expression of miR-342 and ID4 in patients. Negative correlation of ID4 expression measured by qRT-PCR and miR-342 levels detected by miRNA arrays. Samples are represented according to their positive/negative estrogen receptor status. The dashed line was obtained using a linear regression model.

We evaluated the expression levels of ID4 in our cohort of patients by qRT-PCR. We confirmed a negative correlation between ID4 and ESR1 expression (r = −0.62, P<0.0001) and found that ER-positive patients on average had three times smaller ID4 expression compared to ER-negative patients (95% CI: 1.6 to 5.8, P = 0.002) (Figure 2). In our samples, miR-342 and ID4 had opposite expression compared to estrogen receptor, suggesting an interplay among them. In fact, a negative correlation between miR-342 array and ID4 expression data was observed in our cohort of cases (r = −0.44, P = 0.002) (Figure 3). Association of ID4, ESR1 and miR-342 was very similar also in the TCGA data set (Figures S3 and S4).

**Figure 4:**
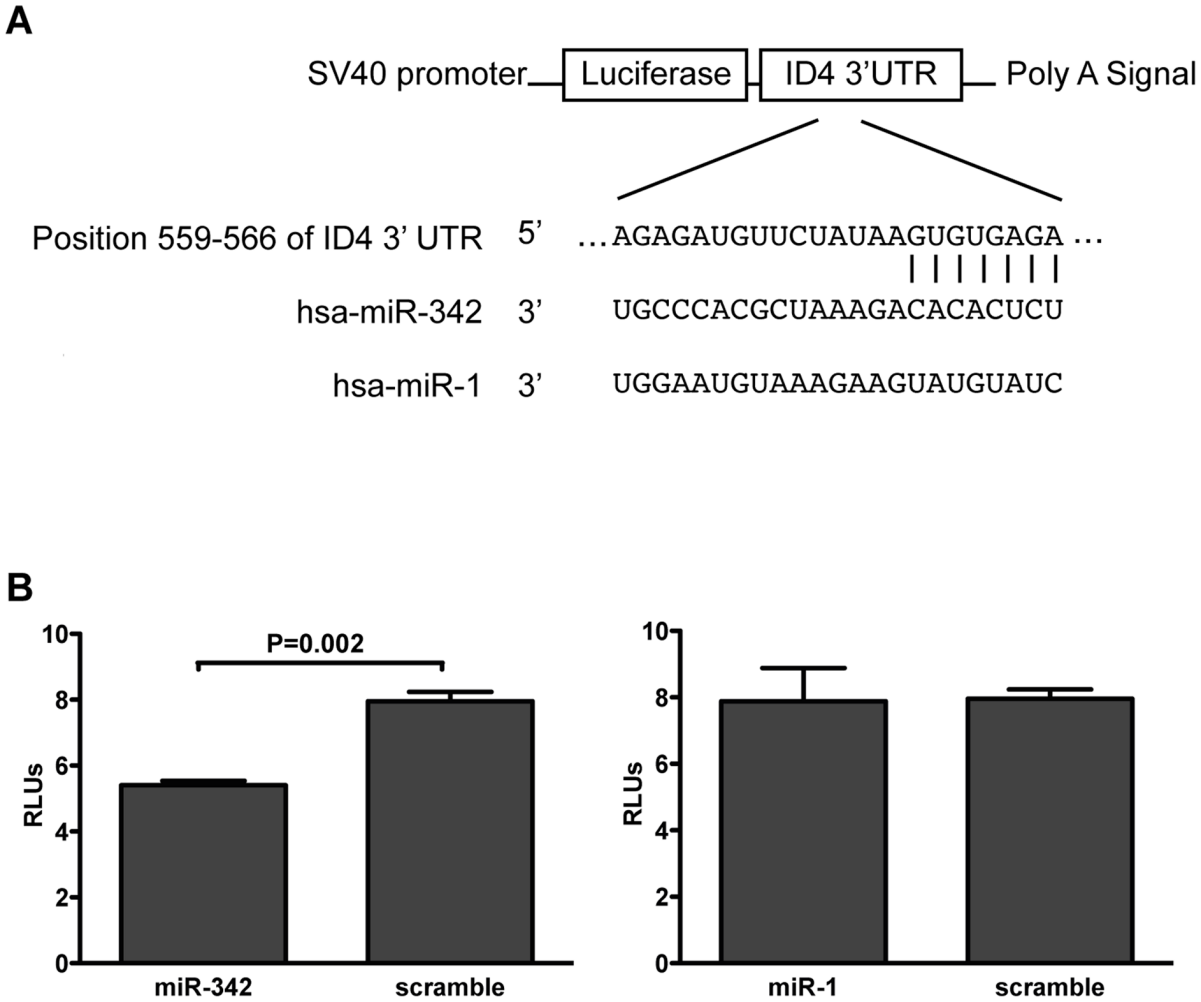
miR-342 directly regulates ID4. (A) The 3′UTR of ID4 gene containing the predicted miR-342 binding site was cloned into pGL3-promoter vector. This construct was cotransfected with precursor molecules of miR-342 or of miR-1 (whose seed region has no affinity for ID4 3′ UTR). (B) Relative luciferase activity in 293T cells was determined after transfection. A scramble oligonucleotide was used as negative control of transfection.

In our data the association between ID4 and miR-342 was attenuated when the analysis was adjusted for ESR1 expression: in the linear regression models with ID4 used as a dependent variable, the regression coefficient of miR-342 was −0.69 (95% CI: −1.1 to −0.27, P = 0.002) in the univariable model, and −0.31 (95% CI: −0.71 to 0.10, P = 0.12) in the model adjusted for the expression of ESR1. A similar result was observed also in the TCGA data set, where the association between ID4 and miR-342 was attenuated but it remained statistically significant in the ESR1 adjusted analysis (P = 0.008, regression coefficients −0.35 and −0.16 in univariable and adjusted analysis, respectively). To functionally validate miR-342 binding to the 3′UTR of the ID4 gene and its potential inhibitory effect on this gene, an *in vitro* model was constructed using a reporter luciferase system. The resulting vector, pLuc ID4-3′UTR, contained the 3′UTR region of ID4 gene with the predicted miR-342 binding site downstream the Luciferase gene (Figure 4A). pLuc ID4-3′UTR vector was cotransfected in 293T cells either with miR-342 precursor molecule, another precursor molecule containing a seed region that does not bind to ID4 3′UTR (miR-1) or a scramble oligonucleotide. Sequences of miR-1 used as aspecific miRNA and of the scramble oligonucleotide were both used as negative controls. As shown in Figure 4B, miR-342 overexpression significantly decreased the luciferase activity compared to the scramble oligonucleotide (P = 0.002). By contrast, transfection of pLuc ID4-3′UTR construct with miR-1 precursor, which has no predicted binding sites for ID4 3′UTR, prevented the down-regulation of luciferase expression. This result indicated that the reduction of luciferase activity was miR-342 specific and that there was a direct interaction between miR-342 and ID4 gene.

### Correlation between ID4 and BRCA1 Expression through miR-342

**Figure 5:**
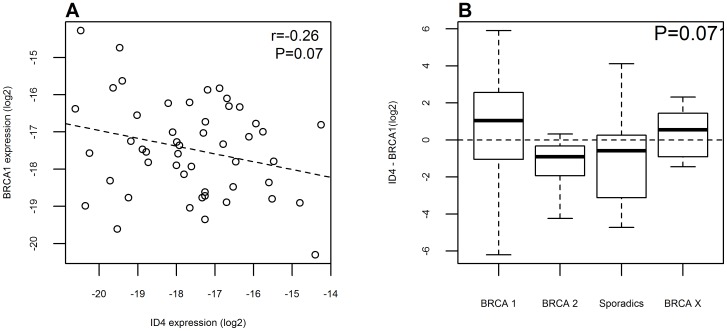
Expression of ID4 and BRCA1 in patients. (A) Negative correlation of ID4 expression and BRCA1 levels both measured by qRT-PCR analysis. The dashed line was obtained using a linear regression model. (B) Differences between delta Ct values of ID4 and BRCA1 in patients divided into hereditary (BRCA1- or BRCA2-mutant), familial (BRCAx) or sporadic groups.

As ID4 can regulate the transcription of BRCA1 [25], we investigated a possible association between the expression levels of these two genes in our cohort of patients. Using a qRT-PCR approach, we observed a negative correlation between ID4 and BRCA1 gene expression (r = −0.26, P = 0.07) in our case series (Figure 5A). A similar negative correlation was observed also in the TCGA data set (r = −0.28, P<0.0001, Figure S5A). Classification of samples according to their sporadic or heredo-familial status (BRCA1- or BRCA2-mutant and BRCAx) showed a different trend of expression of the two genes. BRCA1-mutant and BRCAx cases had higher expression of ID4 and lower expression of BRCA1 compared to sporadic and BRCA2-mutant samples (P = 0.07, Figure 5B). We observed a similar pattern also in the TCGA data set (Figure S5B, P = 0.0013).

Having showed that ID4 is a direct target of miR-342, we hypothesized that modulation of ID4 operated by miR-342 could provide a mechanism for regulating BRCA1 expression.

**Figure 6:**
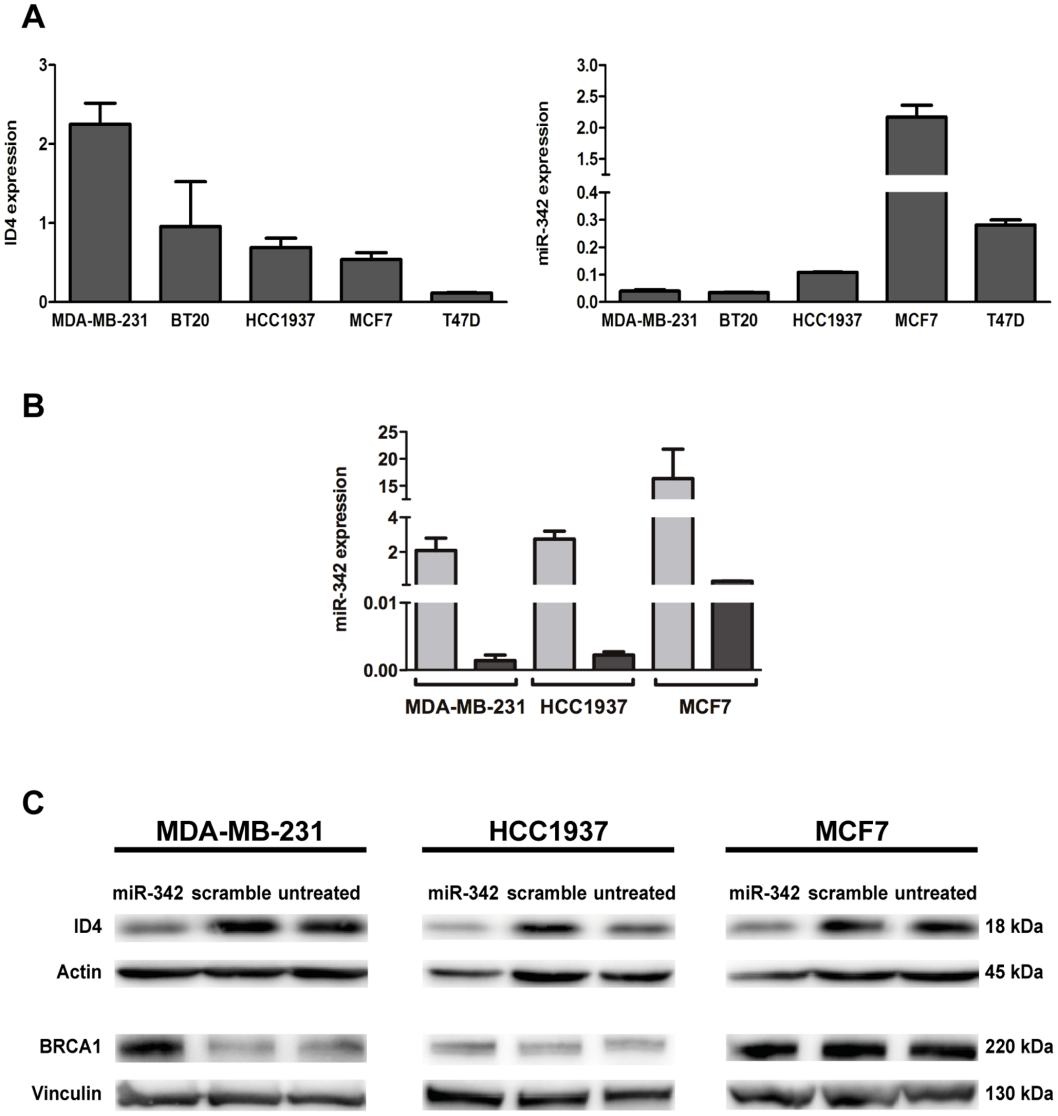
Down-regulation of ID4 protein through miR-342 increases BRCA1 expression in MDA-MB-231 cells. (A) Expression of ID4 (left) and miR-342 (right) measured by qRT-PCR in five breast cancer cell lines. (B) qRT-PCR evaluation of miR-342 expression in MDA-MB-231, HCC1937 and MCF7 cells after transfection of pre-miR-342 (grey) or of a scramble oligonucleotide (black). (C) Western blotting analysis of ID4 and BRCA1 proteins on the same cells. qRT-PCR data are expressed as two elevated to –ΔCt (delta cycle threshold) value which is directly related to the expression levels. β-Actin and Vinculin were used as loading control.

To asses in an *in vitro* model of breast cancer the miRNA-target interaction and its effect on BRCA1, expression levels of ID4 and miR-342 were measured in five human breast cancer cell lines including MDA-MB-231 and BT20 (ER-negative), HCC1937 (BRCA1-mutant, ER-negative) [26], MCF7 and T47D (ER-positive). As shown in Figure 6A, an inverse correlation of ID4 and the miRNA was observed; in particular ID4 gene expression was higher in the ER-negative cell lines. By contrast, miR-342 had higher expression in ER-positive cells. This was in accordance with literature data that report both ID4 and miR-342 expression associated with the ER status of human breast cancer cells [13], [24], [27].

Among the five cell lines analyzed for ID4 and miR-342 expression, we selected MDA-MB-231(ER-negative), HCC1937 (BRCA1-mutant and ER-negative) and MCF7 (ER-positive) cells and transfected them with miR-342 precursor molecule, or with a scramble oligonucleotide, to assess the expression modulation of ID4 and BRCA1 proteins through Western blotting analysis. Overexpression of miR-342, verified in all the three cell lines by qRT-PCR (Figure 6B), led to a reduction of 60% of ID4 protein and to an increase of about 50% of BRCA1 protein in MDA-MB-231 cells compared to cells transfected with a scramble oligonucleotide (Figure 6C) thus confirming that miR-342 can indirectly modulate BRCA1 expression. Reduction of ID4 protein (about 50%) was observed also in HCC1937 cells, which carry both the BRCA1 truncated alleles, but it was not associated with increase of BRCA1 protein. In the MCF7 cells the weak reduction of ID4 protein observed (about 20%) did not lead to an increase of BRCA1 protein (Figure 6C).

**Figure 7:**
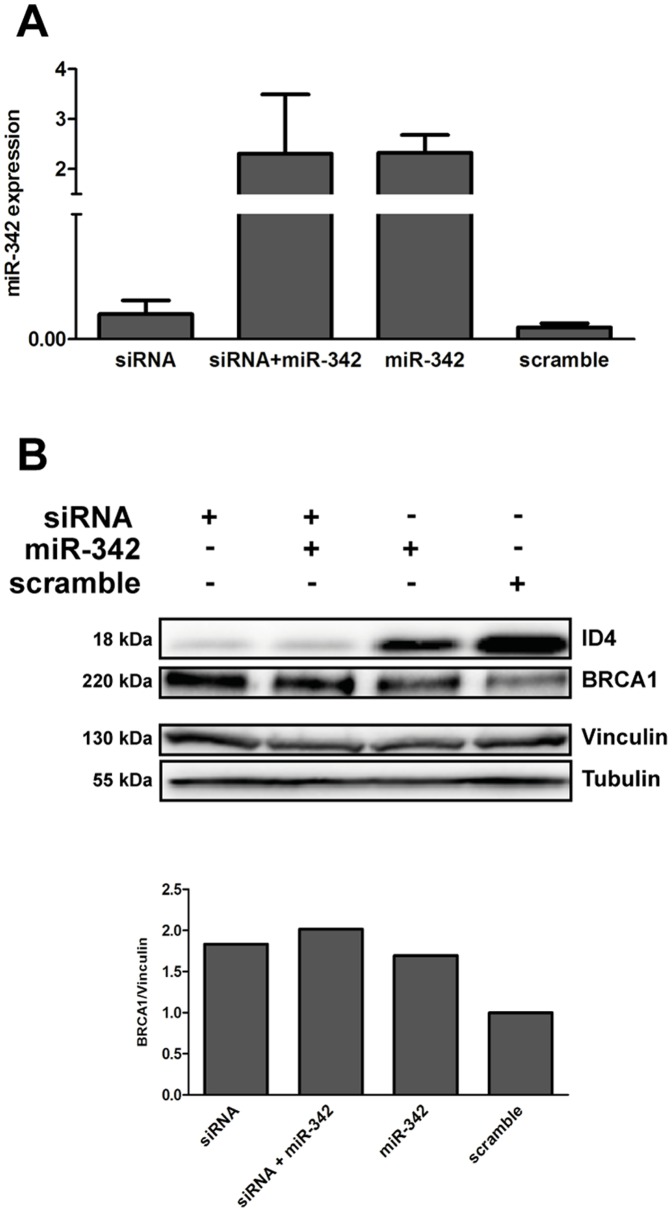
Silencing of ID4 in MDA-MB-231 cells. Transfection of siRNA ID4 and pre-miR-342 or of a scramble oligonucleotide in MDA-MB-231 cells: (A) qRT-PCR evaluation of miR-342 expression. (B) Western blotting analysis of ID4 and BRCA1 proteins; α**-**Tubulin and Vinculin were used as loading control. The figure illustrates one representative experiment and shows the quantified protein expression of BRCA1 normalized to Vinculin. qRT-PCR data are expressed as two elevated to –ΔCt (delta cycle threshold) value which is directly related to the expression levels.

MDA-MB-231 cells transfected with a pool of four oligonucleotides targeting different portions of ID4 (siRNA ID4) that silenced ID4 expression showed increased expression of BRCA1 that was similar to that observed after miR-342 overexpression; this effect was stronger when cells were cotransfected with siRNA ID4 and miR-342 (Figure 7).

### Associated Expression of miR-342 and BRCA1 in Estrogen Receptor Negative Samples

**Figure 8:**
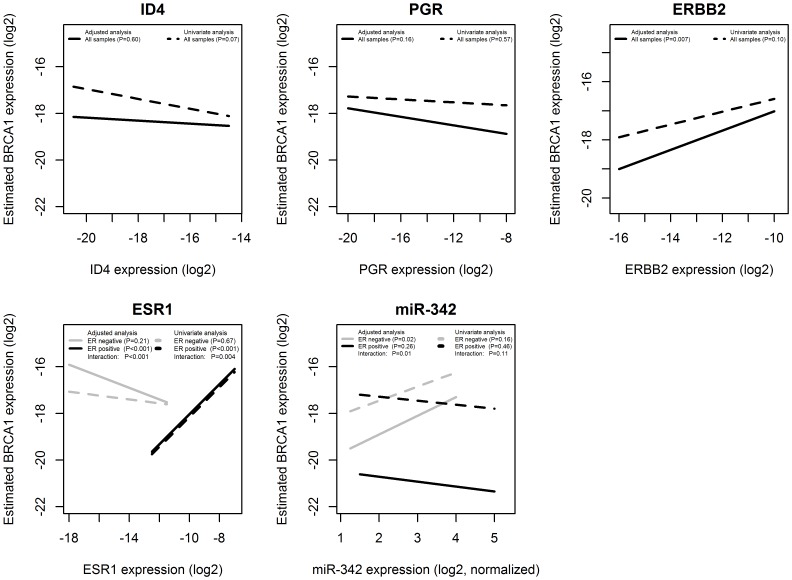
Estimated association between BRCA1 and ID4, PGR, ERBB2, ESR1 and miR-342. The associations were estimated from regression models where BRCA1 was the outcome (y-axis) while the other genes (ID4, PGR, ERBB2, ESR1) and miR-342 were the covariates (x-axis). In the multivariate model (adjusted analysis, showed in solid lines) all the covariates and the interaction terms between ER status and miR-342 were included. The univariate models (showed in dashed lines) included only the specified gene (ID4, PGR, ERBB2, ESR1) or miR-342 as covariates. The analysis of the association of BRCA1 with ER and miR-342 was done dividing patients according to their ER status.

We investigated if the interaction between miR-342 and ID4 resulting in BRCA1 modulation validated *in vitro* could be also observed in the clinical samples included in our case series. To this aim, we assessed if the association of ID4 and miR-342 with BRCA1 was independent from ESR1, PGR and ERBB2. We fitted a multivariable linear regression model in which the BRCA1 gene expression was modelled as a function of ID4, PGR, ERBB2, ESR1 and miR-342; an interaction between ER status and miR-342 expression was also included (adjusted analysis) while the other interaction terms were not statistically significant and were not included. The results were also compared to those obtained using models with only one covariate included (univariariate analysis) as graphically presented in Figure 8. After adjusting the analysis for all the other variables included in the model, statistically there was no significant association between BRCA1 and ID4 (P = 0.60) or PGR (P = 0.16), while an association between BRCA1 and ERBB2 was observed (P = 0.007). The expression of BRCA1 and ESR1 was significantly and positively associated in the group of the ER-positive samples (P<0.001), but not in the group of the ER-negative samples (P = 0.21), even though, on average, the ER-negative and ER-positive samples had similar BRCA1 expression. miR-342 was positively associated to the BRCA1 gene within the subset of ER-negative patients (P = 0.02), while the association was not statistically significant for ER-positive patients (P = 0.26). This finding suggests the existence of a mechanism different from ER status that regulates BRCA1 expression in ER-negative samples that could be operated through expression of miR-342.

Estimating the same model with the TCGA data set we found that ID4 remained statistically significantly associated to BRCA1 in the analysis adjusted for all the other variables (P<0.001), while the positive association between ESR1 and BRCA1 within the ER-positive patients, observed in the univariable analysis, was not statistically significant in the adjusted analysis. The positive association between miR-342 and BRCA1 within the ER-negative patients, observed in the model based on our data, was not confirmed on the TCGA data set (see Figure S6 for complete results).

## Discussion

In this study we demonstrated *in vitro* that a mechanism involving the microRNA miR-342 and the two genes ID4 and BRCA1, which are frequently altered in breast cancer, could have a role in the pathogenesis of this tumor type. We showed in the ER-negative breast cancer cell line MDA-MB-231 that miR-342 indirectly controls the expression of BRCA1 gene through direct regulation of ID4. This result was supported by the finding that, using a siRNA based strategy to reduce ID4 expression, we observed increased expression of BRCA1, comparable to that found with miR-342 transfection.

miR-342 is expressed in several cancers including leukemia [28], breast and colorectal cancer, where it exerts a tumor suppressor function that is operated through induction of apoptosis [29], [30]. In breast cancer, expression of miR-342 has been associated with better prognosis [15], [31]. This finding is supported by the observation that miR-342 is highly expressed in ER-positive tumors, in particular in the luminal molecular subclass of breast cancers [13], [14] characterized by better prognosis, and that its expression is directly correlated with the expression levels of the estrogen receptor [14], [32], [33]. In addition, miR-342 is down-regulated in breast tumors resistant to Tamoxifen, a selective ER modulator commonly used for treating ER-positive breast cancers. Reintroduction of miR-342 sensitizes refractory breast tumor cells to tamoxifen therapy, suggesting that miR-342 is an important regulator of tamoxifen response [34], [35]. In our cohort of breast cancer cases and in five breast cancer cell lines, as well as in an independent *in silico* data set of 283 breast cancer retrieved from TCGA [22], we confirmed the strong positive association between miR-342 expression and ER status, as miR-342 was more highly expressed in ER-positive than in ER-negative cases and cell lines. In order to identify the genes controlled by miR-342 that could be involved in breast cancer, thus underlining the role of miR-342 in this tumor type, we used a computational approach for predicting miRNA target transcripts and identified ID4 as a putative target of miR-342 in all the three databases of miRNA target prediction we investigated. ID4 is a member of the ID (inhibitor of DNA binding) family of the helix-loop-helix-transcription factors (HLH) that do not possess a DNA binding domain. ID proteins function as dominant-negative regulators of basic-helix-loop-helix transcription factors through the formation of inactive heterodimers [36]. The four members of the ID proteins family (ID-1 to 4) are regulators of crucial cellular processes. Increased expression of ID proteins has been reported in several cancer types [37], [38], [39] and has been found associated with loss of differentiation, enhanced malignancy and aggressive clinical behavior [36], [40]. ID4 is involved in mammalian embryogenesis, angiogenesis and in the maintenance of cancer stem cells [41], [42], [43]. In breast cancer, ID4 expression is quite controversial; in fact, it was found in normal breast epithelium, and, while it was not detected in ER-positive tumors or neoplastic lesions [24], it was present in ER-negative tumors [27]. Beger and colleagues showed that an increase in ID4 expression was associated with the ability of breast cancer cells to exhibit anchorage-independent growth, while its depletion determined their morphological change to large and flat epithelial phenotypes [25]. The finding that ID4 is a negative regulator of the tumor suppressor gene BRCA1 [25], [27], [44] supports an oncogenic function of ID4. Germline mutations of BRCA1 predispose to breast cancer and are characteristic of the hereditary form of this tumor [6]. Somatic mutations in this gene are very rare in the sporadic form, while decreased expression levels of BRCA1 have been identified in the triple negative group, or basal-like subtype, of sporadic breast cancers (ER, PR and HER2 negative) that have genetic profiles similar to BRCA1 mutation carrier tumors [44] and express high levels of ID4 [45]. We confirmed that ID4 expression is inversely correlated to ER levels and observed an inverse correlation between ID4 and expression of miR-342 in our cohort of cases, in the independent data set and in the breast cancer cell lines we analyzed, which supported the interaction between miR-342 and ID4 predicted *in silico*. Finally, our *in vitro* experiments conducted using Luciferase assays, functionally confirmed that ID4 is a direct target of miR-342. This miRNA could therefore be involved in the modulation of ID4 in breast cancer. When we evaluated mRNA levels of ID4 and BRCA1 in our cohort of cases and in the validation set, we observed a negative association of their expression that suggested a role for the possible interaction of the two genes in the pathogenesis of breast cancer, possibly through miR-342 mediated regulation of ID4 that modulates BRCA1. microRNA operated regulation of BRCA1 has already been reported in another study where miR-335 was shown to target four different modulators of BRCA1 expression, including ID4 [46]. However, interaction of ID4 with miR-335 was very weak compared with that of the other BRCA1 modulators (ERα, IGF1R and SP1) and no reduction of Luciferase activity was shown with the ID4 construct. Most likely, the effect of miR-335 on BRCA1 expression was operated through the other three targets. Our study shows that miR-342 directly binds ID4, as demonstrated by the Luciferase reporter assay, and the BRCA1 modulation we observed in the cellular model was actually provoked by the reduction of ID4 as ERα, IGF1R and SP1 are not predicted targets of miR-342. Wen et al. found that triple negative breast cancers present high levels of ID4 protein respect to the other breast cancers and propose that overexpression of ID4 could functionally inactivate BRCA1 in this tumor type which presents characteristics similar to BRCA1-mutant tumors and downregulation of BRCA1 [45]. In the case series we analyzed, we observed a correlation between miR-342 and BRCA1 expression for the ER-negative cases that were mostly also PGR- and ERBB2- negative. However, the fact that most of them were also BRCA1-mutant cases has so far prevented us to demonstrate a direct link between cancer and reduced expression of the gene. The correlation between miR-342 and BRCA1 was not confirmed in the independent data set, where the ER-negative series also included non PGR- and ERBB2-negative cases. However, the fact that the inverse correlation between ID4 and miR-342 and ID4 and BRCA1 remained statistically significant highlights that the interplay between the miRNA and the two genes could exist. On the other hand, we successfully demonstrated the interplay between miR-342 and the two genes in MDA-MB-231 cells that share the features of the clinical “triple negative” tumor type for their ER, PR and HER2 negative status, and have been classified in the basal B subtype based on their transcriptional profile [47], [48]. The reason why we chose this triple negative cell line was that it expressed high levels of ID4 compared with the other triple negative cell lines analyzed. Moreover, because of its endogenous low expression of miR-342, it represented a good model where to do the overexpression of miR-342 and to study its effects on ID4 and BRCA1 interaction. To support our hypothesis, overexpression of miR-342 in MCF7 cells, that are ER-positive and present high levels of miR-342 and BRCA1 but low ID4 expression, only resulted in a weak reduction of ID4 that has no effects on BRCA1 expression. We also evaluated changes in the expression of ID4 and BRCA1 in the BRCA1-mutant cell line HCC1937 and found that transfection of miR-342 strongly reduced ID4, but not BRCA1 expression. However in these cells ID4 and miR-342 are expressed at lower levels than in MDA-MB-231 that perhaps are not enough for activating the mechanism of BRCA1 modulation operated by ID4.

The fact that the cohort of patients we analyzed also included heredo-familial cases prevented us from observing a direct effect of miR-342 on BRCA1 activity since most of the ER-negative cases analyzed were BRCA1-mutant. However, these samples had higher expression of ID4 and lower expression of BRCA1 compared to sporadic and BRCA2-mutant cases, and the same correlation for BRCA1 and ID4 was observed for the BRCAx group. This observation suggests that at least part of the familial predisposition to breast cancer of the heterogeneous BRCAx group could be explained by a regulatory effect of ID4 on BRCA1, possibly involving miR-342. However, we can only speculate that miR-342 has a role in the reduced expression of BRCA1, since all the BRCAx cases we analyzed where ER-positive (and this miRNA is lower in an ER-negative context). To validate this hypothesis, measurement of expression levels of miR-342, ID4 and BRCA1 and the assessment of their correlation should be extended to a larger cohort of BRCAx samples including ER-negative cases. Results from larger investigations could clarify if miR-342 can be considered an additional player in the genetic predisposition to breast cancer.

## Supporting Information

Figure S1 **Association of individual miRNA expression differences between samples and ER, PR and HER2 status.** Estimated regression coefficients of the miRNAs obtained from the regression models were significantly associated to ER, PR and HER2 status (for details, see the Methods section). Positive numbers indicate higher miRNA expression levels in the groups ER, PR and HER2 positive. Only the miRNAs with P value from at least one regression model <0.10 are displayed. Note that the expression of the miRNAs was log-2 transformed.
(TIFF)

Figure S2 **miR-342 expression according to ESR1 expression levels or ER status in the TCGA data set.** (A) Positive correlation of miR-342 and ESR1 expression levels. The dashed line was obtained using a linear regression model. (B) Box plot representation of miR-342 expression according to estrogen receptor negative or positive status of the patients analyzed.
(TIFF)

Figure S3 **ID4 expression according to ESR1 expression levels or ER status in the TCGA data set.** (A) Negative correlation of ID4 and ESR1 expression levels. The dashed line was obtained using a linear regression model. (B) Box plot representation of ID4 expression considering estrogen receptor negative or positive status of the patients analyzed.
(TIFF)

Figure S4 **Expression of miR-342 and ID4 in the TCGA data set.** Negative correlation of ID4 and miR-342 expression levels. Samples are represented according to their positive/negative estrogen receptor status. The dashed line was obtained using a linear regression model.
(TIFF)

Figure S5 **Expression of ID4 and BRCA1 in the TCGA data set.** (A) Negative correlation of ID4 and BRCA1 expression levels. The dashed line was obtained using a linear regression model. (B) Differences between expression of ID4 and BRCA1 in patients divided into hereditary (BRCA1- or BRCA2-mutant) or sporadic groups.
(TIFF)

Figure S6 **Estimated association between BRCA1 and ID4, PGR, ERBB2, ESR1 and miR-342 in the TCGA data set.** The associations were estimated from regression models where BRCA1 was the outcome (y-axis) while the other genes (ID4, PGR, ERBB2, ESR1) and miR-342 were the covariates (x-axis). In the multivariate model (adjusted analysis, showed in solid lines) all the covariates and the interaction terms between ER status and miR-342 were included. The univariate models (showed in dashed lines) included only the specified gene (ID4, PGR, ERBB2, ESR1) or miR-342 as covariates. The analysis of the association of BRCA1 with ER and miR-342 was done dividing patients according to their ER status.
(TIFF)

Table S1 **Clinical and histopathological characteristics of breast cancer samples.** BrCa: Breast cancer; Contr BrCa: Controlateral BrCa; Ipsi BrCa: Ipsilateral BrCa; OvCa: Ovarian cancer; Brca in Italic: tumoral samples used for microarray analysis; IDC: infiltrating ductal carcinoma; ADK: adenocarcinoma; ILC: infiltrating lobular carcinoma; Gel: gelatinous; pT: tumor dimension, according to the AJCC staging criteria; pN: number of positive lymph nodes/total lymph nodes removed, according to the AJCC staging criteria; P: axillary dissection from previous BrCa; *histology after primary chemotherapy for previous BrCa; B/O familiarity for Breast/Ovarian cancer; CNS: Central Nervous System; uk: unknown; nd: not determined; Ned: Not evident disease.
(XLSX)

Table S2 **miRNAs associated with estrogen, progesterone and human epidermal growth factor 2 receptor.** Positively/negatively associated miRNAs expression was in positive/negative correlation to the expression or status of estrogen receptor, progesterone receptor and human epidermal growth factor receptor 2 (P<0.05). Bold type is used for the miRNAs that were significantly associated with the status (*) and the expression (**) of the genes. For details on the analysis see the materials and methods section. Two different miR-321 sequences originated from two different miRNA precursors had a statistically significant association with ERBB2 expression.
(XLSX)
