# Supplementary figures and images for: miR-342 Regulates BRCA1 Expression through Modulation of ID4 in Breast Cancer
Source: PLoS One. 2014 Jan 27;9(1):e87039. doi: 10.1371/journal.pone.0087039 (PMC3903605; doi:10.1371/journal.pone.0087039)

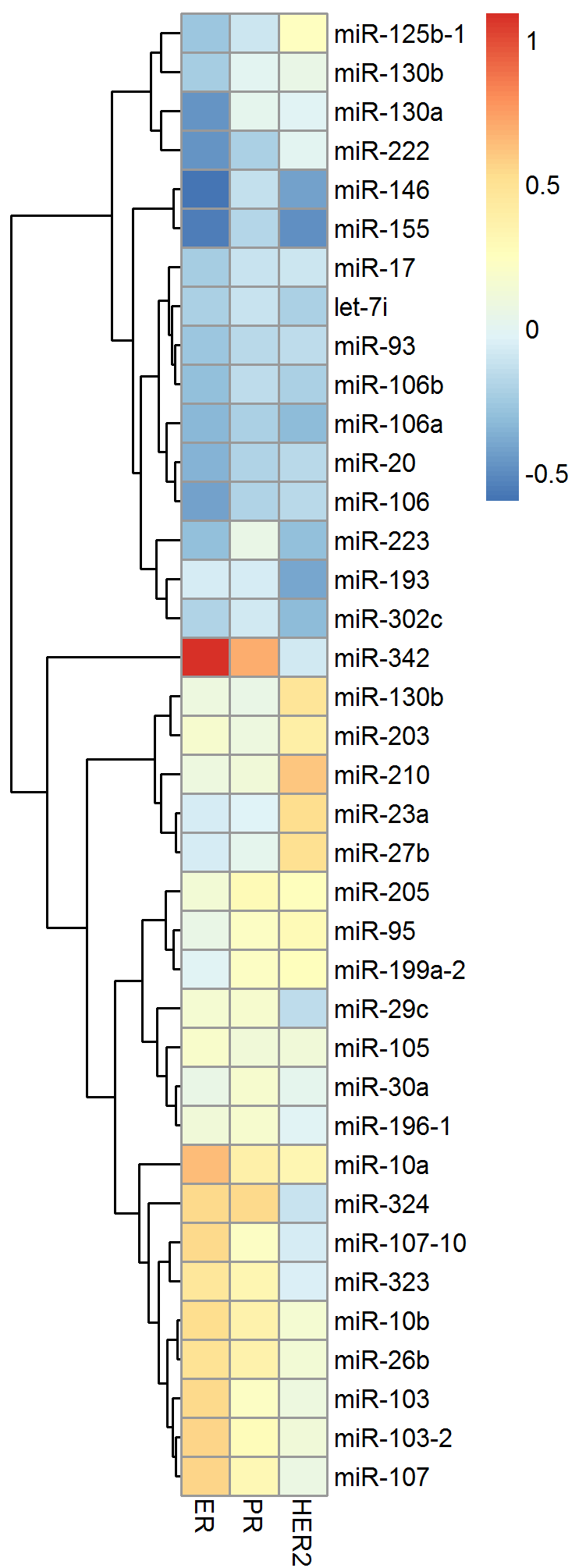

Supplement: Figure S1 — Association of individual miRNA expression differences between samples and ER, PR and HER2 status. Estimated regression coefficients of the miRNAs obtained from the regression models were significantly associated to ER, PR and HER2 status (for details, see the Methods section). Positive numbers indicate higher miRNA expression levels in the groups ER, PR and HER2 positive. Only the miRNAs with P value from at least one regression model <0.10 are displayed. Note that the expression of the miRNAs was log-2 transformed. (TIFF) [file pone.0087039.s001.tiff]

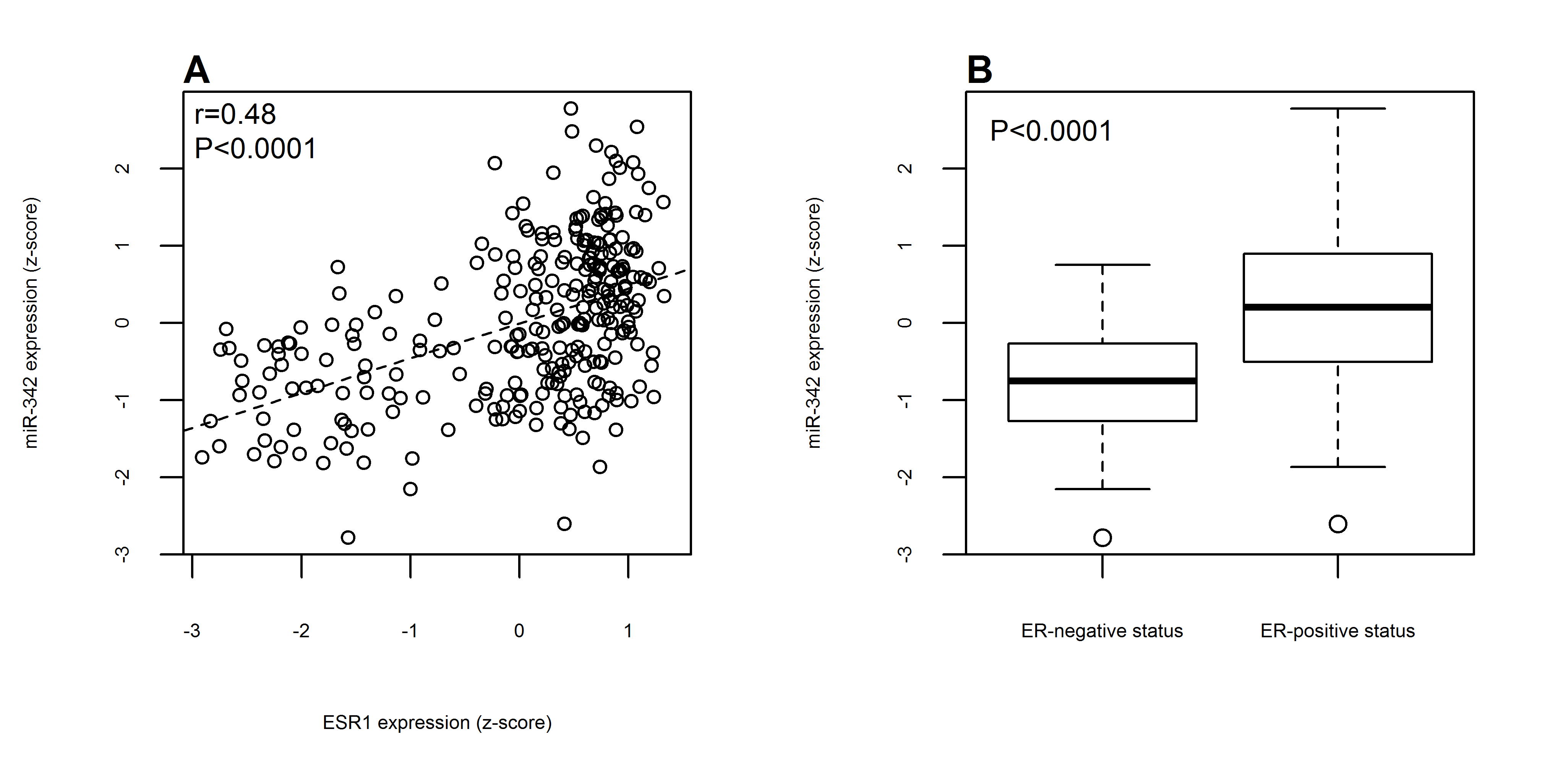

Supplement: Figure S2 — miR-342 expression according to ESR1 expression levels or ER status in the TCGA data set. (A) Positive correlation of miR-342 and ESR1 expression levels. The dashed line was obtained using a linear regression model. (B) Box plot representation of miR-342 expression according to estrogen receptor negative or positive status of the patients analyzed. (TIFF) [file pone.0087039.s002.tiff]

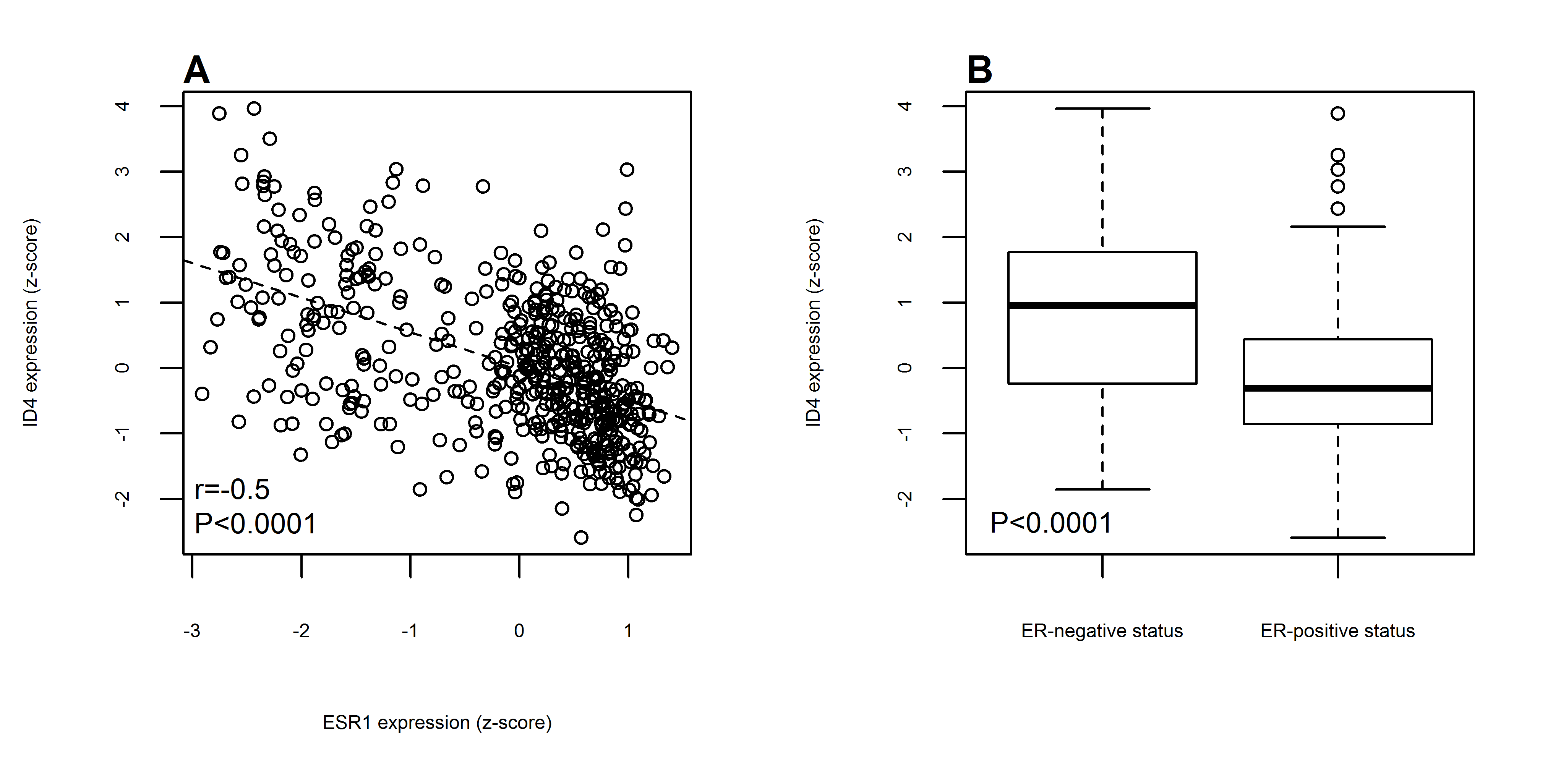

Supplement: Figure S3 — ID4 expression according to ESR1 expression levels or ER status in the TCGA data set. (A) Negative correlation of ID4 and ESR1 expression levels. The dashed line was obtained using a linear regression model. (B) Box plot representation of ID4 expression considering estrogen receptor negative or positive status of the patients analyzed. (TIFF) [file pone.0087039.s003.tiff]

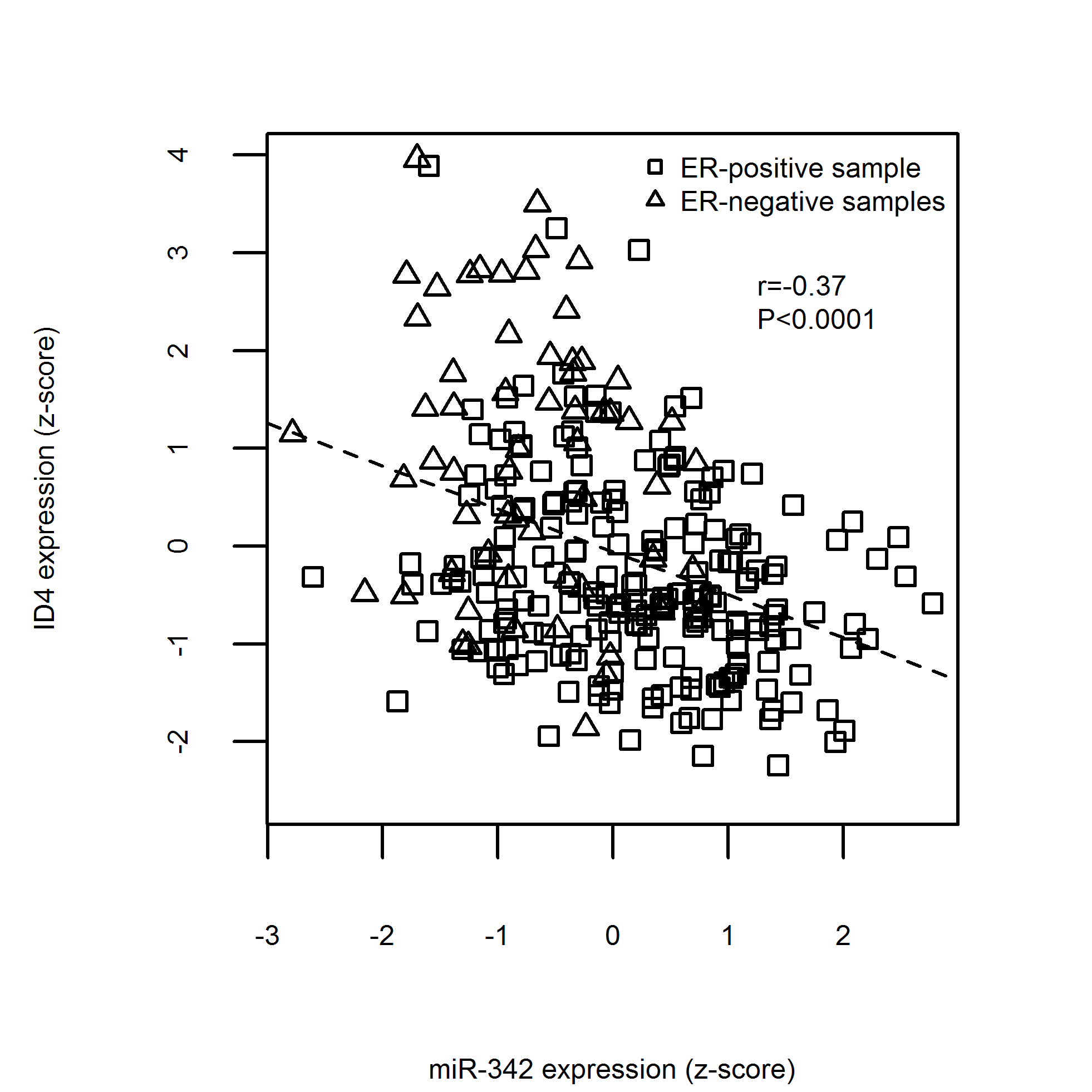

Supplement: Figure S4 — Expression of miR-342 and ID4 in the TCGA data set. Negative correlation of ID4 and miR-342 expression levels. Samples are represented according to their positive/negative estrogen receptor status. The dashed line was obtained using a linear regression model. (TIFF) [file pone.0087039.s004.tiff]

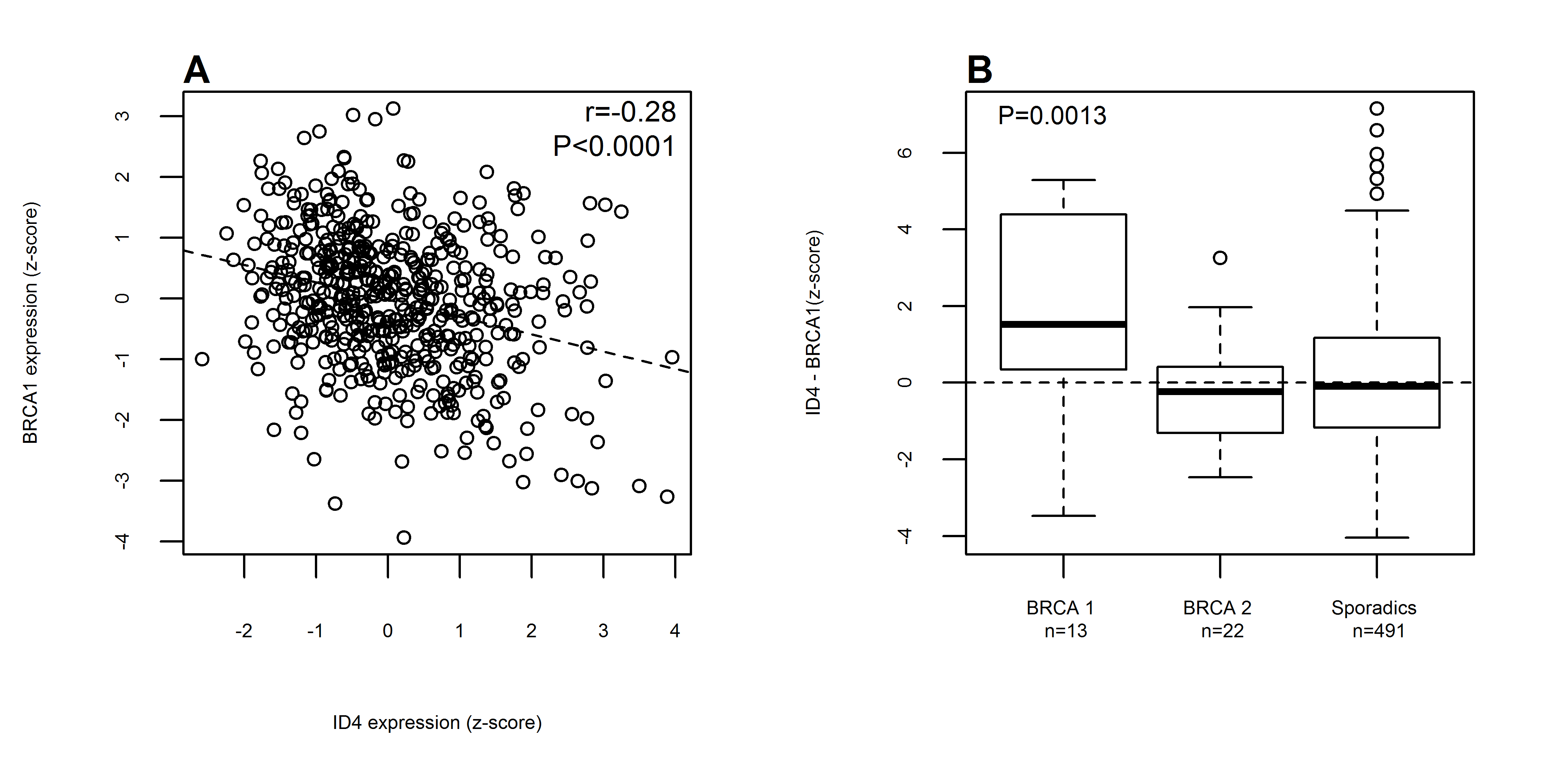

Supplement: Figure S5 — Expression of ID4 and BRCA1 in the TCGA data set. (A) Negative correlation of ID4 and BRCA1 expression levels. The dashed line was obtained using a linear regression model. (B) Differences between expression of ID4 and BRCA1 in patients divided into hereditary (BRCA1- or BRCA2-mutant) or sporadic groups. (TIFF) [file pone.0087039.s005.tiff]

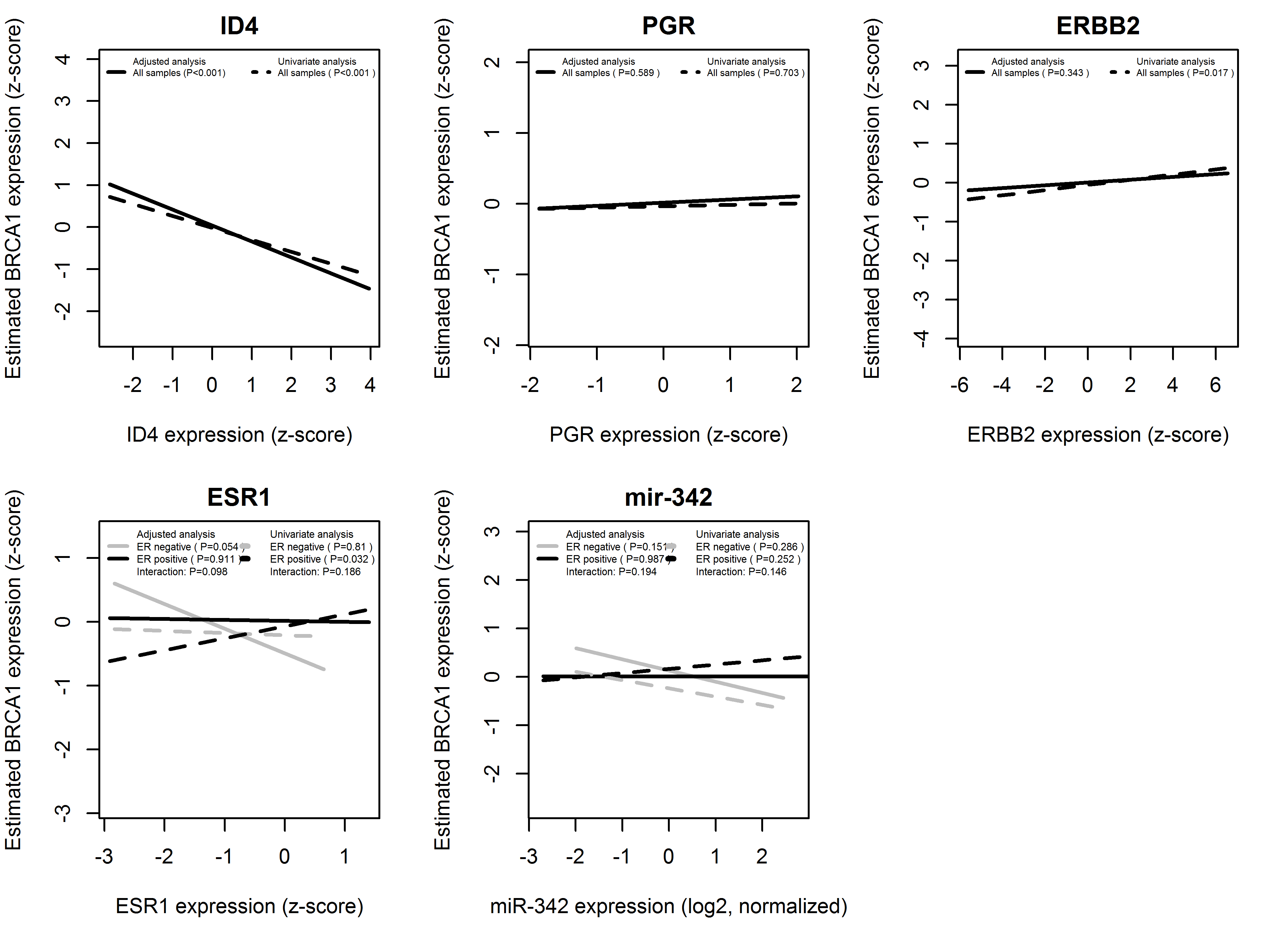

Supplement: Figure S6 — Estimated association between BRCA1 and ID4, PGR, ERBB2, ESR1 and miR-342 in the TCGA data set. The associations were estimated from regression models where BRCA1 was the outcome (y-axis) while the other genes (ID4, PGR, ERBB2, ESR1) and miR-342 were the covariates (x-axis). In the multivariate model (adjusted analysis, showed in solid lines) all the covariates and the interaction terms between ER status and miR-342 were included. The univariate models (showed in dashed lines) included only the specified gene (ID4, PGR, ERBB2, ESR1) or miR-342 as covariates. The analysis of the association of BRCA1 with ER and miR-342 was done dividing patients according to their ER status. (TIFF) [file pone.0087039.s006.tiff]
